# A comparative analysis of methodological practices used in capture–recapture population size estimation: opioid use disorder prevalence in New York State, 2020

**DOI:** 10.1093/aje/kwag161

**Published:** 2026-07-10

**Authors:** Heather Bradley, Trang M Nguyen, Lanxin Li, Ruth King, Vivian Kamanu, Serveh Sharifi Far, Stephanie Mack, Nicole Luisi, Yunshu Li, Yali Meng, Gang Liu, Jiaji Zhang, Ashly E Jordan, Linh Le, Peter L Brodie, Svetlana Jensen, Allan Clear, Tomoko Udo, Vanda Inacio, Eli S Rosenberg

**Affiliations:** Department of Epidemiology, Emory University Rollins School of Public Health, 1518 Clifton Rd. NE, Atlanta, GA 30322, United States; Office of Science, New York State Department of Health, Empire State Plaza, Albany, NY 12237, United States; Department of Epidemiology and Biostatistics, College of Integrated Health Sciences, University at Albany, 1400 Washington Avenue, Albany NY 12222, United States; School of Mathematics and Maxwell Institute for Mathematical Sciences, University of Edinburgh, James Clerk Maxwell Building, The King’s Buildings Campus, Peter Guthrie Tait Road, Edinburgh EH9 3FD, United Kingdom; School of Mathematics and Maxwell Institute for Mathematical Sciences, University of Edinburgh, James Clerk Maxwell Building, The King’s Buildings Campus, Peter Guthrie Tait Road, Edinburgh EH9 3FD, United Kingdom; Office of Science, New York State Department of Health, Empire State Plaza, Albany, NY 12237, United States; School of Mathematics and Maxwell Institute for Mathematical Sciences, University of Edinburgh, James Clerk Maxwell Building, The King’s Buildings Campus, Peter Guthrie Tait Road, Edinburgh EH9 3FD, United Kingdom; Office of Science, New York State Department of Health, Empire State Plaza, Albany, NY 12237, United States; Department of Epidemiology, Emory University Rollins School of Public Health, 1518 Clifton Rd. NE, Atlanta, GA 30322, United States; Office of Science, New York State Department of Health, Empire State Plaza, Albany, NY 12237, United States; Office of Science, New York State Department of Health, Empire State Plaza, Albany, NY 12237, United States; Office of Science, New York State Department of Health, Empire State Plaza, Albany, NY 12237, United States; Office of Science, New York State Department of Health, Empire State Plaza, Albany, NY 12237, United States; Research and Program Evaluation, New York State Office of Addiction Services and Supports, New York, NY, United States; Office of Science, New York State Department of Health, Empire State Plaza, Albany, NY 12237, United States; Division of State Emergency Medical Services, New York State Department of Health, 875 Central Avenue, Albany, NY 12206, United States; Bureau of Narcotic Enforcement, New York State Department of Health, 150 Broadway, Menands, NY 12204, United States; Office of Drug User Health, New York State Department of Health, 90 Church Street, New York, NY 10007, United States; Department of Epidemiology and Biostatistics, College of Integrated Health Sciences, University at Albany, 1400 Washington Avenue, Albany NY 12222, United States; Department of Health Policy, Management, and Behavior, College of Integrated Health Sciences, University at Albany, 1400 Washington Avenue, Albany, NY 12222, United States; School of Mathematics and Maxwell Institute for Mathematical Sciences, University of Edinburgh, James Clerk Maxwell Building, The King’s Buildings Campus, Peter Guthrie Tait Road, Edinburgh EH9 3FD, United Kingdom; Office of Science, New York State Department of Health, Empire State Plaza, Albany, NY 12237, United States; Department of Epidemiology and Biostatistics, College of Integrated Health Sciences, University at Albany, 1400 Washington Avenue, Albany NY 12222, United States

**Keywords:** capture–recapture, opioid use disorder (OUD), data linkage, Bayesian statistics

## Abstract

Prevalence estimates for opioid use disorder (OUD) are essential for guiding investments in prevention and treatment. Previous estimates have been produced using modeling approaches including capture–recapture (CRC). Empirical evaluations of CRC are needed to assess population size impacts of methodological practices utilized. We linked administrative data sources including New York State (NYS) adults with OUD by indication between January 1 and December 31, 2020 and applied Bayesian CRC methods to evaluate how methodological practices impact OUD prevalence: (1) using sensitive vs specific case definition characteristics to identify OUD, (2) including vs excluding inferred referrals from emergency medical services to healthcare settings, and (3) including vs excluding overdose mortality as a source list. We estimated OUD prevalence overall and by demographic characteristics and treatment coverage with medications for OUD (MOUD). Across methodological scenarios, OUD prevalence estimates ranged from 4.6% to 9.9%. The largest estimate resulted from a model in which the more sensitive OUD case definition was applied, while the smallest resulted from a model using the more specific case definition. An estimated 4.2% of NYS adults had OUD in 2020, with 20% receiving MOUD. Findings demonstrate that CRC methodological decisions can meaningfully influence OUD prevalence estimates, with recommended methodological practices provided.

## Introduction

The US opioid crisis, now in its third decade, began with extensive use of prescribed opioids, evolved to include heroin use, and since 2016 has been driven primarily by illicit use of synthetic opioids. During 1999-2023, more than 800 000 people died of opioid overdose.^1^ Many more have experienced opioid-related morbidity, including opioid use disorder (OUD), which the *DSM*-5 defines as “a problematic pattern of opioid use leading to clinically significant impairment or distress.”^2^^,^^3^ Recent reductions in opioid overdose mortality are encouraging, with rates declining over the last several years.^4^ However, the extent to which OUD burden has changed in recent years is largely unknown because OUD prevalence is difficult to measure at a population level. A better understanding of OUD prevalence is essential for increasing public investments in care and treatment.^5^

There are a limited number of US-based OUD prevalence estimates. Because survey data yield underestimates for substance use outcomes due to household sampling methods and reliance on self-report,^6-8^ previous estimates are derived from modeling.^3^^,^^9-19^ Several modeling approaches have been used, but 1 of the most robust is capture–recapture (CRC), also known as multiple systems estimation.^9^^,^^16-18^ CRC methods use probability principles to estimate the unobserved size of a target population through detection of person-level overlap across combined datasets, that is, source lists. The utilization of multiple, incomplete lists of population members is ideal for estimating the prevalence of a substance use disorder for which many people are undiagnosed and under-represented in routine clinical data. However, CRC methods rely on a number of assumptions, including a closed population in which there are no deaths, births, or migration during the estimation period; independence of source lists; and equal individual probabilities of appearing on each source list.^20^ Although analytical tools can address some assumption violations (eg, interaction terms in log-linear models to relax the source list independence assumption), assumptions pose challenges that are often specific to target populations and source lists.

CRC has potential for more widespread use in estimating prevalence of substance use outcomes in the United States. However, empirical evaluations of CRC applications to US data are needed to assess the extent to which there are population size impacts of commonly used methodological practices, including some regarding key model assumptions. First, OUD case definitions used by previous US CRC studies minimally require only 1 opioid overdose or opioid-related encounter to meet criteria for a capture in some source lists.^9^^,^^16^^,^^17^ A single opioid overdose may not be indicative of OUD for multiple reasons, including fentanyl contamination of non-opioid substances^21-23^ and first-time or occasional opioid use that may not meet OUD diagnostic criteria.^2^ Sub-optimal specificity of this case definition may lead to overestimation of OUD prevalence. Second, previous studies have not addressed possible referrals of OUD patients from emergency medical services (EMSs) to hospital-based source lists.^9^^,^^16^^,^^17^ Including referrals across source lists, where a single OUD encounter is considered to be 2 captures, may bias estimates due to violation of the source list independence assumption, which may not be fully addressed using interaction term.^24^ In the case of OUD prevalence estimation, such referrals may happen commonly as individuals experiencing opioid overdose are often transported by EMS to hospitals.^25^ Lastly, previous studies have included opioid-related overdose deaths as captures using vital statistics (VS) data.^9^^,^^16^^,^^17^ Including such mortality data violates the “closed population” assumption, requiring a more careful interpretation of results and parameter estimates.^26^

In this manuscript, we use linked administrative data from New York State (NYS) and CRC methods to accomplish 3 objectives.

Objective 1: To evaluate OUD population size estimation impacts of 3 CRC methodological practices: (1) use of a sensitive vs specific case definition for identifying OUD in source lists, (2) inclusion vs exclusion of duplicate captures representing inferred referrals from EMS to healthcare settings, and (3) inclusion vs exclusion of overdose mortality data as a source list.

Objective 2: To apply CRC in estimating OUD prevalence among adults aged 18+ years in NYS in 2020, stratified by region, sex, and age group using a Bayesian modeling averaging approach.

Objective 3: To estimate treatment coverage with medications for opioid use disorder (MOUD) and the opioid overdose mortality: OUD population size ratio, overall and within demographic strata.

## Methods

### Data sources, linkage, and OUD case definitions

We used 4 NYS data sources for these analyses: VS, prescription monitoring program (PMP), EMSs, and the Statewide Planning and Research Cooperative System (SPARCS). Each person was assigned a within-data source unique pseudo-ID based on name, sex, and date of birth. First, OUD case definitions were applied to each source list, and only pseudo-IDs meeting case definitions in each source list were retained for linkage. Other variables retained in source lists were sex, age, region (NYC and Rest of State [ROS]), and data fields (including dates) that were used to determine OUD case status. Each pseudo-ID was retained only once for matching, using information from the first date the case definition was met. Then, linkage across source lists was implemented using exact matching by pseudo-ID. Analyses for this study were then performed with the deidentified linked data.

The 2 OUD case definitions used are described in Table 1. Case definition 1 was derived from previous US-based CRC studies, originating with Barocas et al.^9^ OUD case definition 1 requires a minimum of 1 opioid-related encounter (eg, overdose), and OUD case definition 2 requires a minimum of 2 opioid-related incidents. For both, this criterion applies in the absence of documented OUD (ie, through use of an International Classification of Diseases [ICD]-10 code).

**Table 1:** Data sources and case definitions used to classify opioid use disorder (OUD) for capture–recapture analysis, New York State, 2020.

**Data source**	**Description**	**Population**	**Case definition 1**	**Case definition 2**
**Emergency medical services (EMSs)**	Emergency medical service pre-hospital care reports from licensed ambulance services in NYS. The reports consist of patient demographics, incident details, clinical information, and EMS provider actions	All persons receiving EMSs pre-hospital care in NYS	One or more opioid-related incident(s)^a^	1. Evidence of treatment for OUD or opioid dependence/abuse/misuse in EMS narrative or complaint OR 2. Evidence of IV opioid use in EMS narrative or complaint OR 3. At least 2 encounters with F11 or T40 ICD-10-CM code in primary or secondary impression OR 4. Repeated opioid use observed for at least 2 encounters in EMS narrative or complaint OR 5. Two or more suspected opioid overdose encounters
**Prescription monitoring program (PMP)**	Filled prescription data for controlled substances in schedules II-V reported by all dispensing pharmacies and practitioners registered with NYS and pharmacies outside of NYS that dispense 600 prescriptions or more a year to NYS residents. The data consist of patient’s demographic and controlled substance prescription information	All persons with a filled prescription for schedule II-IV medications from all NYS community, hospital outpatient, and clinic pharmacies and from out-of-state mail order pharmacies that deliver to patients in NYS	Filled prescription for buprenorphine for OUD treatment	Filled prescription for buprenorphine for OUD treatment
**Statewide Planning and Research Cooperative System (SPARCS)**	Repository of hospital discharge data from facilities regulated by New York State. Discharge stays and visits are collected across 4 main claim types—inpatient, emergency department, ambulatory surgery, and outpatient services. Information collected includes patient characteristics, diagnoses and treatments, services, and charges. Diagnoses are coded using ICD-10-CM codes	All persons receiving inpatient and outpatient care from NYS hospitals	1. Any inpatient or outpatient encounter with an ICD-10-CM discharge diagnostic code (any diagnosis) containing F1110, F1111, F11120, F11121, F11122, F11129, F1113, F1114, F11150, F11151, F11159, F11181, F11182, F1118, F1119, F1120, F1121, F11220, F11221, F11222, F11229, F1123, F1124, F11250, F11251, F11259, F11281, F11282, F11288, F1129, F1190, F1191, F11920, F11921, F11922, F11929, F1193, F1194, F11950, F11951, F11959, F11981, F11982, F11988, F1199 for opioid use disorder 2. Any opioid overdose resulting in an inpatient or outpatient encounter indicated by ICD-10-CM diagnostic code (any diagnosis): T40.0, T40.1, T40.2, T40.3, T40.4, T40.6 (excludes “adverse effect” or “underdosing” as indicated by the values of 5 and 6 in the 6th character; and “sequela” as indicated by the value of “S” in the 7th character; eg, T400X5S, T400X6S) without regard to intent	1. Any inpatient or outpatient encounter with an ICD-10-CM discharge diagnostic code (any diagnosis) containing F1110, F1111, F11120, F11121, F11122, F11129, F1113, F1114, F11150, F11151, F11159, F11181, F11182, F1118, F1119, F1120, F1121, F11220, F11221, F11222, F11229, F1123, F1124, F11250, F11251, F11259, F11281, F11282, F11288, F1129, F1190, F1191, F11920, F11921, F11922, F11929, F1193, F1194, F11950, F11951, F11959, F11981, F11982, F11988, and F1199 for opioid use disorder 2. Two or more opioid overdoses resulting in an inpatient or outpatient encounter within a calendar year indicated by ICD-10-CM diagnostic code (any diagnosis): T40.0, T40.1, T40.2, T40.3, T40.4, and T40.6 (excludes “adverse effect” or “underdosing” as indicated by the values of 5 and 6 in the 6th character; and “sequela” as indicated by the value of “S” in the 7th character; eg, T400X5S, T400X6S) without regard to intent
**Vital statistics of New York State (VS)**	Deathr NYS residents, which include detailed demographic, clinical, and circumstantial information on the decedent	All deceased NYS residents	Opioid-related overdose deaths with ICD codes or word searches on literal causes of death excluding suicides	NA

Abbreviations: ICD, International Classification of Diseases; NA, not available; NYS, New York State; OUD, opioid use disorder.

a Adapted from Barocas et al.^9^

Analyses included persons aged 18+ years with OUD by indication, for example, identified by either OUD case definition in any of the 4 datasets during January 1-December 31, 2020. Hereafter, these persons with inferred OUD will be referred to as “people with OUD.”

### Deduplication of referrals

To compare OUD prevalence estimates in scenarios where referrals were removed (ie, deduplication across source lists applied) vs not removed (ie, deduplication not applied), referrals assumed to represent the same opioid-related encounter were deduplicated between EMS, SPARCS, and VS as follows. Opioid overdose deaths identified in EMS or SPARCS and in VS were removed from VS and counted in either EMS or SPARCS, wherever they were first observed. If a person was observed with an opioid overdose in EMS and later observed in SPARCS within 2 days, the SPARCS encounter was removed. For case definition 2, the total number of events was then recounted to determine eligibility for meeting the SPARCS OUD case definition. For persons identified with OUD in EMS encounters and later discharged from a hospital (SPARCS data) with a primary diagnosis code for OUD, the SPARCS encounter was removed.

### Contingency tables

We categorized individual data by region NYC and ROS, age group (18-29, 30-64, and 65+ years), and sex (male and female) for stratification in the contingency tables. Using linked source lists, contingency tables were created by summarizing the number of people with OUD in each source list conditional on their OUD status in other source lists (Table S1). When sex was missing (~0.01%), it was assigned via probabilistic imputation, with probability equal to the observed proportion within sex, region, and age group strata.

Contingency tables were created as follows; stratified by region, sex, and age group. For Objective 1, we compared OUD prevalence estimates among adults aged 18-64 years to align with other US-based CRC studies,^16-18^ facilitating easier comparisons across them, and also to avoid parameter estimation problems due to observed zeros introduced by the joint use of the VS source list and adults aged 65+ years. We compared prevalence estimates using 2 alternatives for each of 3 methodological practices. We assessed each practice in all combinations with alternatives from the other 2 and, as such, created 8 (2^3^) sets of contingency tables. *Case definition 1*: (A1) all source lists; (B1) SPARCS, PMP, and EMS; (C1) SPARCS, EMS, and VS deduplicated for referrals, PMP; (D1) SPARCS and EMS deduplicated for referrals, PMP. *Case definition 2*: (A2) all source lists, (B2) SPARCS, PMP, and EMS; (C2) SPARCS, EMS, and VS deduplicated for referrals, PMP; (D2) SPARCS and EMS deduplicated for referrals, PMP.

For Objective 2, we created a single contingency table for persons aged 18+ years using SPARCS and EMS source lists, deduplicated for referrals, and PMP (Table S2).

### Data analysis

For Objectives 1 and 2, a Bayesian CRC method^26^^,^^27^ was used to generate overall and stratified OUD estimates. We used standard log-linear models^20^^,^^28^ that account for dependence between data sources and/or covariates via inclusion of interaction terms (Appendix S1). Given a specific log-linear model, in terms of interactions present, the posterior distribution for total OUD population size can be obtained by combining the posterior predictive unobserved OUD counts under each possible combination of covariates returned by the model and observed OUD counts. The posterior distribution of the OUD population for specific individual covariate values or combinations can be similarly calculated.

There are many possible log-linear models, depending on which interaction terms are included, therefore model selection is typically required. However, the estimate of the population size can be sensitive to the choice of model. Thus, we consider a model-averaging approach that accounts for both parameter and model uncertainty within a Bayesian framework.^26^^,^^27^^,^^29^

Within the Bayesian model-averaging approach, we consider the set of hierarchical log-linear models^30^ ($\mathcal{M}$). Let $\boldsymbol{\beta} =\left({\beta}_0,{\beta}_1,\boldsymbol{\cdots},{\beta}_q\right)$ denote the parameter vector of length $\left(q+1\right)$ corresponding to the maximal model (the most complex model includes the maximum number of interaction terms) in the set $\mathcal{M}$. For each log-linear model $m\in \mathcal{M}$, the likelihood function is represented as: $\pi \left(\boldsymbol{y}|{\boldsymbol{\beta}}_m,m\right)$, where $\boldsymbol{y}=\left({y}_1,\cdots, {y}_n\right)$ denotes the cell counts corresponding to a contingency table with *n* entries, and ${\boldsymbol{\beta}}_m\subseteq \boldsymbol{\beta}$ denotes the set of parameters in model *m*. The likelihood is then combined with the prior: $\pi \left({\boldsymbol{\beta}}_m,m\right)=\mathrm{\pi} \left({\boldsymbol{\beta}}_m|m\right)\pi (m)$, which reflects our a priori beliefs concerning the parameters and models before observing any data, to form a posterior distribution:


$$ \pi \left(\boldsymbol{\beta} |\boldsymbol{y}\right)={\sum}_{m\in \mathcal{M}}\pi \left({\boldsymbol{\beta}}_m,m|\boldsymbol{y}\right)\propto{\sum}_{m\in \mathcal{M}}\pi \left(\boldsymbol{y}|{\boldsymbol{\beta}}_m,m\right)\mathrm{\pi} \left({\boldsymbol{\beta}}_m|m\right)\pi (m), $$

which represents our updated belief on the parameters ($\boldsymbol{\beta}$) after observing the given data ($\boldsymbol{y}$). Without any strong prior information, uninformative prior distributions were used.

To explore and summarize the posterior distribution, we implement a reversible jump Markov chain Monte Carlo algorithm.^31^ The algorithm explores the parameter and model space simultaneously so that only a single MCMC chain is required. Posterior samples were generated by using the R package *mse*, as well as to estimate both model parameters and the posterior inclusion probability of each possible interaction term. Accordingly, the model-averaged posterior distribution of the total OUD population size can be computed, which we summarize in terms of the posterior mean and the 95% credible interval (CI), accounting for parameter and model uncertainty.

For Objective 3, overall and strata-specific estimated OUD population sizes from Objective 2 and the number of NYS patients receiving methadone^32^ and buprenorphine^33^ for OUD treatment in 2020 were used as denominators and numerators, respectively, for estimation of MOUD treatment coverage. Overdose deaths involving opioids for NYS in 2020 were obtained from the CDC Multiple Cause of Death query^34^ and used as numerators for overall and strata-specific estimation of opioid overdose mortality ratios per 1000 persons with OUD.

Data processing, matching, and aggregation were conducted using SAS statistical software. Statistical analyses were implemented using R software. This study was not considered human subjects research by the NYSDOH Institutional Review Board.

## Results

### Objective 1: comparing the impacts of CRC methodological practices on OUD estimates

Across combinations of methodological practices as applied to NYS data, OUD prevalence ranged from 4.6% (CI, 4.3%-5.0%) to 9.9% (CI, 6.6%-15.7%) (Table 2). The largest estimate resulted from a modeling scenario in which the (sensitive) OUD case definition 1 was applied, referrals were not deduplicated, and overdose mortality data were included as a source list. The smallest estimate was derived from a scenario in which the (specific) OUD case definition 2 was applied, referrals were deduplicated, and mortality data were excluded.

**Table 2:** Capture–recapture estimates of opioid use disorder (OUD) prevalence under methodological practice alternatives, New York State, adults aged 18-65 years, 2020.

**Methodological component**		**Case definition 1** ^**a**^							**Case definition 2** ^**a**^					
		**Observed**	**Unobserved**	**Total estimate OUD**		**Prevalence estimate OUD**			**Observed**	**Unobserved**	**Total estimate OUD**		**Prevalence estimate OUD**	
		***n***	***n***	***n***	**95% CI**	**%**	**95% CI**		***n***	***n***	***n***	**95% CI**	**%**	**95% CI**
Data deduplication not applied Overdose mortality data inclusion	A1	136 417	1 076 491	1 212 908	812 366-1 924 329	9.91	6.63%-15.72%	A2	127 539	537 520	665 059	61 357-719 507	5.43	5.01%-5.88%
Data deduplication not applied Overdose mortality data exclusion	B1	133 767	727 415	861 182	799 972-927 930	7.03	6.53%-7.58%	B2	124 564	506 043	630 607	579 056-689 007	5.15	4.73%-5.63%
Data deduplication applied^b^ Overdose mortality data inclusion	C1	136 395	680 992	817 387	770 387-868 200	6.68	6.29%-7.09%	C2	127 529	656 792	784 321	575 331-894 748	6.41	4.70%-7.31%
Data deduplication applied^b^ Overdose mortality data exclusion	D1	133 744	689 538	823 282	771 448-879 427	6.72	6.30%-7.18%	D2	124 553	440 680	565 233	526 760-606 866	4.62	4.30%-4.96%

Abbreviations: CI, credible interval; EMS, Emergency medical services; OUD, opioid use disorder; SPARCS, Statewide Planning and Research Cooperative System.

a See Table 1 for data sources and definitions.

b Deduplication: between SPARCS/EMS for the same encounter removed cases where individual only captured as OUD in SPARCS if transported to hospital/ED within 2 days after OUD EMS encounter; persons with OUD indicated as dead in EMS or SPARCS and also found in Vital Records OUD data were removed from Vital Records and only counted once in EMS or SPARCS wherever they were first observed.

Under definition 1, there was an appreciable difference in OUD prevalence estimates when referrals were not deduplicated and mortality data were included (9.9%) vs when referrals were deduplicated and mortality data were excluded (6.7%) but with overlapping CIs. Under case definition 2, the difference for the same comparison was smaller (5.4% vs 4.6%), and the CIs were narrowly non-overlapping. All comparisons by case definition resulted in significantly different OUD prevalence estimates (eg, A1 vs A2; 9.9% vs 5.4%) except where referrals were deduplicated and mortality data were included. No significant differences in prevalence estimates were found when comparing inclusion vs exclusion of mortality data alone (eg, A1 vs B1) or non-deduplication vs deduplication of referrals alone (eg, A1 vs C1). Posterior inclusion probabilities for interaction terms included in each model are provided in Table S3, and posterior density estimates for OUD prevalence under each combination of methodological practices are provided in Figure S1.

### Objective 2: estimating OUD prevalence and opioid-related indicators for NYS in 2020

An estimated 4.2% (CI, 3.9%-4.5%) of adults aged 18+ years in NYS had OUD in 2020, corresponding to 648 026 (CI, 604 744-694 752) people (Figure 1, Table S4). Of those, an estimated 441 117 (CI, 408 102-476 354) were in ROS (5.0%, CI, 4.6%-5.4%) and 206 908 (CI, 191 669-223 976) were in NYC (3.1%, CI, 2.9%-3.4%) (Table S4). Prevalence was higher among males (5.4%, CI, 5.1%-5.8%) compared to females (3.1%, CI, 2.8%-3.3%), and this disparity was larger in NYC than in ROS. By age group, prevalence was highest among 30- to 64-year-olds (5.5% statewide, CI, 5.1%-5.9%). The age group with lowest prevalence was 18- to 29-year-olds (1.2%, CI, 1.1%-1.4%) in NYC and 65+ year-olds (2.7%, CI, 2.3%-3.0%) in ROS. Prevalence was nearly double the statewide average among males aged 30-64 years in ROS (7.9%, CI, 7.3%-8.6%, Table S4).

**Figure 1:**
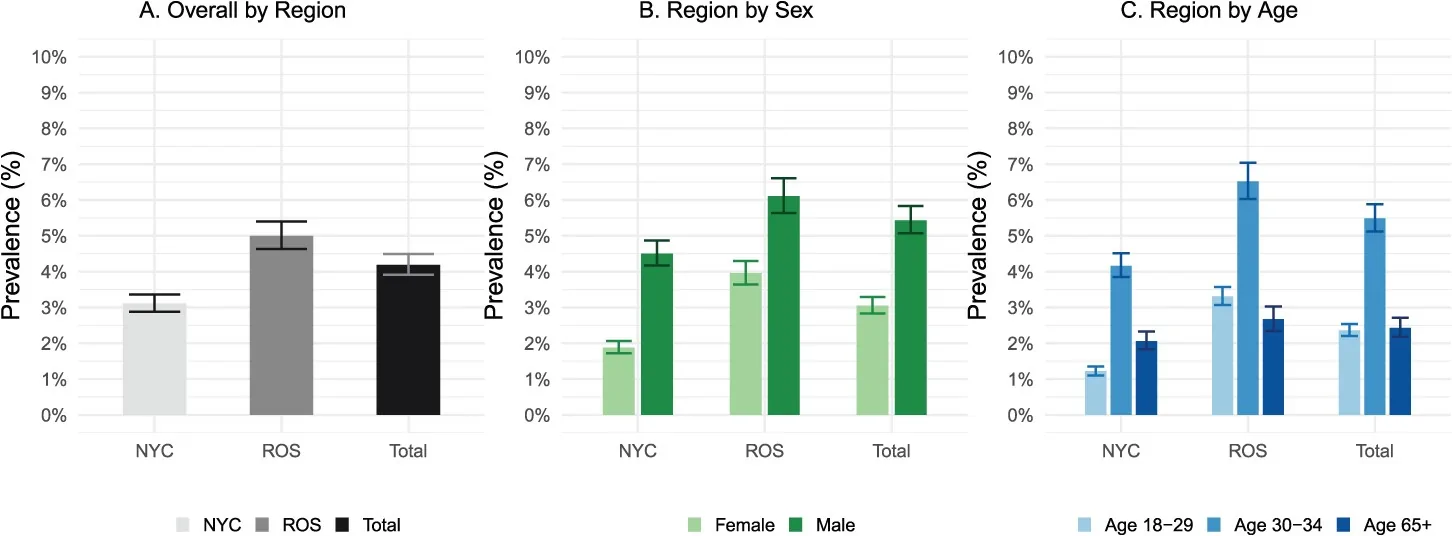
Capture–recapture estimates of opioid use disorder (OUD) prevalence, New York State, adults aged 18+ years, 2020. Abbreviations: NYC, New York City; NYS, New York State; ROS, rest of state.

### Objective 3: estimating treatment coverage with MOUD and the opioid overdose mortality ratio

An estimated 20% (CI, 18.6%-21.4%) of adults with OUD were receiving MOUD in NYS in 2020 (Table 3). This percentage was higher in NYC (23.5%, CI, 21.7%-25.4%) than in ROS (18.3%, CI, 16.9%-19.8%). People aged 65+ years had lower estimated treatment coverage (8.1%, CI, 7.3%-9.1%) compared to those in other age groups. Overall, there were 6.5 opioid overdose deaths per 1000 estimated people with OUD (CI, 6.1-7.0). This ratio was higher in NYC (8.2, CI, 7.6-8.8) compared to ROS (5.7, CI, 5.3-6.2). Males had a higher estimated overdose mortality ratio (7.8, CI, 7.2-8.3) compared to females (4.5, CI, 4.1-4.8). The largest disparity in the overdose mortality ratio was observed by age group, where the youngest age group had the highest ratio (9.2, CI, 8.6-9.9). This disparity was particularly pronounced in NYC, where there were 12.6 opioid overdose deaths per 1000 estimated 18- to 29-year-olds with OUD.

**Table 3:** Estimated treatment coverage with medications for opioid use disorder (MOUD) and opioid overdose deaths per 1000 people with OUD, New York State, adults aged 18+ years, 2020.

**Region**	**Sub-group**	**Estimated OUD population size**		**Percentage receiving MOUD treatment** ^**a**^		**Opioid overdose deaths per 1000 people with OUD**		
		***n***	**95% CI**	***n* (%)**	**95% CI**	***n***	**Ratio per 1000**	**95% CI**
**NYS**	Total	648 026	604 744-694 752	129 302 (20.0%)	18.6%-21.4%	4220	6.5	6.1-7.0
	Age 18-29	77 538	72 255-83 241	18 282 (23.6%)	22.0%-25.3%	716	9.2	8.6-9.9
	Age 30-64	492 119	459 099-527 511	105 034 (21.3%)	19.9%-22.9%	3283	6.7	6.2-7.2
	Age 65+	78 369	70 316-87 360	6372 (8.1%)	7.3%-9.1%	221	2.8	2.5-3.1
	Male	402 424	375 204-431 548	83 239 (20.7%)	19.3%-22.2%	3122	7.8	7.2-8.3
	Female	245 602	227 816-265 076	46 049 (18.7%)	17.4%-20.2%	1098	4.5	4.1-4.8
**NYC**	Total	206 908	191 669-223 976	48 638 (23.5%)	21.7%-25.4%	1690	8.2	7.5-8.8
	Age 18-29	18 047	16 342-19 981	3751 (20.8%)	18.8%-23.0%	227	12.6	11.4-13.9
	Age 30-64	163 019	150 821-176 755	41 512 (25.5%)	23.5%-27.5%	1345	8.3	7.6-8.9
	Age 65+	25 842	22 931-29 206	3678 (14.2%)	12.6%-16.0%	118	4.6	4.0-5.1
	Male	140 392	130 076-152 017	34 574 (24.6%)	22.7%-26.6%	1319	9.4	8.7-10.1
	Female	66 517	60 832-72 908	14 063 (21.1%)	19.3%-23.1%	371	5.6	5.1-6.1
**ROS**	Total	441 117	408 102-476 354	80 660 (18.3%)	16.9%-19.8%	2530	5.7	5.3-6.2
	Age 18-29	59 490	55 128-64 196	14 531 (24.4%)	22.6%-26.4%	489	8.2	7.6-8.9
	Age 30-64	329 100	304 397-355 406	63 518 (19.3%)	17.9%-20.9%	1938	5.9	5.5-6.4
	Age 65+	52 527	46 183-59 678	2694 (5.1%)	4.5%-5.8%	103	2.0	1.7-2.2
	Male	262 032	241 915-283 532	48 664 (18.6%)	17.2%-20.1%	1803	6.9	6.4-7.5
	Female	179 085	164 792-194 606	31 983 (17.9%)	16.4%-19.4%	727	4.1	3.7-4.4

Abbreviations: CI, credible interval; MOUD, medications for opioid use disorder; NYC, New York City; NYS, New York State; OUD, opioid use disorder; ROS, rest of state.

a Data source: NYS OASAS (methadone treatment) and NYS PMP (buprenorphine treatment).

## Discussion

Variation in these 3 CRC methodological practices may meaningfully impact population size results. Excluding overdose mortality data as a source list and deduplicating referrals may impact prevalence estimates, particularly in combination and under a more specific case definition. However, estimates differed with non-overlapping CIs in nearly every scenario where sensitive vs specific OUD case definitions were contrasted, suggesting specificity of the OUD case definition is the most important of the 3 elements assessed in terms of impact on estimates.

In previous US-based CRC studies, the OUD case definition required persons to experience minimally 1 opioid-related incident during the estimation period.^9^^,^^16^^,^^17^ We posit a more specific case definition may be needed for several reasons. First, multiple substances are often implicated in overdoses, particularly stimulants and opioids,^35^^,^^36^ where opioid contamination may be a concern.^21-23^ In NYS, the percentage of overdose deaths involving cocaine with synthetic opioids other than methadone, a proxy for fentanyl, increased from 22% in 2015 to 76% in 2020.^25^ Second, there is evidence that a single opioid overdose may not correlate well with diagnosed OUD.^37-40^ Third, the *DSM-5* requires multiple criteria to be met for OUD diagnosis.^2^ Estimation of treatment needs should reflect diagnostic criteria to the extent possible.

For NYS estimates, we elected to deduplicate referrals from EMS to SPARCS despite overlapping CIs for estimates when referrals were deduplicated vs not. Prior studies suggest deduplication of referrals across source lists as best practice in CRC analyses.^24^ The inclusion of interaction terms in log-linear models permits incorporation of dependencies across source lists.^20^ However, in the case of referrals from one source list to another, interaction terms may not provide an adequate model solution, resulting in biased population size estimates.^24^ We therefore recommend deduplicating referrals at the data linkage stage where possible. We also excluded mortality data as a source list for NYS estimates despite overlapping CIs under conditions of inclusion vs exclusion. Inclusion of these data as a source list violates the “closed population” CRC assumption, rendering captured individuals ineligible for recapture.^20^^,^^28^ This introduces complexity both in terms of the interpretation of the model parameters because one’s ability to be observed is conditional on death, and population size estimates. A recent CRC application from Massachusetts noted similar concerns regarding use of overdose mortality data and excluded these data in a sensitivity analysis.^17^ We recommend not using mortality data as a source list without further analytic consideration, given complexities in interpretation introduced by violation of the “closed population” assumption.

Of note, CIs for all estimates under case definition 1 overlap due to the wide A1 interval, which is explained by inclusion of an interaction term for SPARCS^*^PMP^*^EMS. Models including this interaction term had wide CIs. This interaction term was identified in more than half the candidate models for the A1 analysis but not identified elsewhere (Table S3). This dependency may be partially explained by the combination of a non-specific case definition and non-deduplication of referrals. Notably, when the specific case definition, deduplication of referrals, and exclusion of mortality data were used together, the point estimate (D2) differed substantially from the A1 estimate.

Bayesian modeling is another important difference between our application of CRC methods and previous US-based applications, which employed classical modeling approaches. Bayesian modeling has several advantages over classical modeling for CRC. Rather than relying on a single “best” candidate model, the Bayesian approach averages estimates across multiple competitive models weighted by fit criteria.^26^^,^^27^ This reduces bias resulting from selection of a model that may not represent optimal fit, or may be overfit to data in the context of complex interactions.^26^^,^^27^^,^^41^ Additionally, rather than modeling each demographic stratum separately and summing results, as was done in previous applications, we modeled all data simultaneously. This allows incorporation of interaction terms between demographic variables as needed, permitting borrowing of information across strata, resulting in a better model fit and presentation of multiply-stratified results.

The NYS OUD prevalence estimates suggest substantial disparities by region, sex, and age. Males, persons residing in ROS, and those aged 30-64 years had the greatest OUD burden. Estimated OUD denominators allow consideration of disparities in burden alongside disparities in treatment coverage and mortality risk. For example, ROS had higher OUD prevalence but lower treatment coverage compared to NYC. In NYC, the youngest age group had lowest OUD prevalence and highest opioid overdose mortality: OUD ratio. Enumerated opioid overdose deaths suggest highest mortality risk among 30- to 64-year-olds, but OUD denominators allow estimation of a ratio that shows highest risk among the youngest age group. Findings suggest treatment expansion is needed, particularly for ROS, and that overdose mortality prevention efforts should include young people, particularly in NYC.

Previous studies have estimated OUD prevalence in NYS using diverse methods. Zheng et al. estimated 4.6% OUD prevalence among adults aged 18-64 years in ROS counties during 2017-2019 using a Bayesian synthesis approach with unlinked data sources.^19^ Our 2020 estimate for ROS was similar (5.0%), but this estimate would have been slightly higher excluding the oldest age group. Santaella-Tenorio et al. estimated ~2.2% opioid misuse in NYS in 2018.^15^ In this study, self-reported opioid misuse prevalence was model-allocated to counties. Anchoring on National Survey on Drug Use and Health (NSDUH) data likely explains much of the difference between our estimates.^6-8^ Last, Krawczyk et al. estimated 345 730 persons aged 12+ had OUD in NYS during 2019,^13^ nearly 47% lower than our estimate. In this study, a multiplier (4.5×), based on the ratio of OUD prevalence, derived from NSDUH vs CRC in Massachusetts, was applied to every state’s NSDUH estimate. This multiplier likely varies considerably across states, which may account for much of the observed difference.

Although methodological details can be debated, US-based CRC studies, originating with Barocas et al.’s work in Massachusetts (2018),^9^ have moved the substance use field forward considerably in our understanding of opioid-related morbidity. CRC methods have now been used to estimate OUD in at least 3 US states^9^^,^^16^^,^^17^ while maintaining data security and confidentiality standards. Expansion of CRC in the United States for substance use outcomes will require health departments to use surveillance data in new ways including person-level linkage across datasets and possibly partnerships with academic institutions. The innovative CRC work that has been done to date—through health department and academic partnerships—serves as a model for research with tangible policy and programmatic impacts and capacity to move epidemiological methods forward meaningfully.

This study has several limitations. First, we included buprenorphine prescriptions from PMP but did not have individual-level data for methadone treatment or non-prescription treatment episodes. CRC studies in Massachusetts included data on OUD treatment admissions, methadone retention, and from children born with neonatal abstinence syndrome.^9^^,^^17^ Including such data may have resulted in a larger OUD prevalence estimate for NYS, but this limitation is not expected to diminish inferences from the methodological comparisons. Second, we used pseudo-IDs based on names, sex, and date of birth to link persons across datasets. There is a possibility that some matches were misclassified given the large NYS population size. Third, the specific OUD case definition used for NYS is subject to false negatives, just as the sensitive case definition yields false positives. Given the direction of trends in polysubstance use and fentanyl contamination of the drug supply, we assess that for OUD population size estimation, the former limitation is less of a threat to information bias than the latter.

Lastly, the opioid overdose mortality: estimated OUD population size ratios should be interpreted cautiously. If the probability that decedents had OUD differs by demographic characteristics, comparisons across strata will be biased. Because we lack information about undiagnosed OUD among decedents, we do not know the extent or direction of this bias. For example, young decedents may be less likely than older decedents to have OUD and may die due to inexperience using opioids. Alternatively, older decedents may be less likely than younger decedents to have OUD due to survival bias associated with the probability of premature death due to OUD. Importantly, the ratios represent an upper bound of the mortality rate among persons with OUD and underscore needs for interventions that prevent opioid-related death.

We demonstrated that methodological practices appreciably influence estimates generated by CRC models. There was nearly a 650 000-person difference in the estimated population size with OUD when we simultaneously contrasted 3 practices. There are, however, implementation factors to consider when considering use of the analytic strategies recommended here. For example, deduplicating inferred referrals is somewhat resource intensive. Our findings suggest that where this is infeasible, it is better to create OUD estimates without deduplicating referrals than not to produce estimates. Additionally, overdose mortality data are one of the most valuable sources of information about opioid-related risk in the United States, and this information is lost when they are excluded from CRC analyses. Innovative analytic methods are needed to use these data appropriately in CRC studies.

Estimates of OUD prevalence are critical for strategic allocation of resources and monitoring of prevention and treatment intervention effectiveness over time. These estimates are particularly important at state and local levels, where policy and programmatic decisions are made. OUD denominators are essential for illuminating disparities in prevalence of OUD alongside disparities in treatment coverage and mortality risk. US-based CRC studies have shown OUD can be estimated robustly while preserving data confidentiality standards. Here, we offer several methodological adjustments with potential to improve CRC estimates. Further methods development is needed to reduce the impact of violating CRC model assumptions. Public health and academic partnerships, together with creative uses of surveillance data, can facilitate CRC applications and help end the US opioid crisis.

## Supplementary Material

Web_Material_kwag161

## Data Availability

This study was a secondary analysis of surveillance data from the NY SPARCS, EMSs, PMP, and VS. These data are not available for re-release.
