# Supplementary material for: A comparative analysis of methodological practices used in capture–recapture population size estimation: opioid use disorder prevalence in New York State, 2020
Source: Am J Epidemiol. 2026 Jul 10;195(11):2950–8. doi: 10.1093/aje/kwag161 (PMC13733501; doi:10.1093/aje/kwag161)
Supplement: Supplementary file 1 [file web_material_kwag161.docx]

**Supplementary Material**

**A comparative analysis of methodological practices used in capture-recapture population size estimation: Opioid use disorder prevalence in New York State, 2020**

Heather **Bradley**, PhD ^1^, Trang M **Nguyen**, MD, DrPH, MPH ^2,3^, Lanxin **Li**, PhD ^4^, Ruth **King**, PhD ^4^, Vivian **Kamanu**, MPH ^2^, Serveh **Sharifi Far**, PhD ^4^, Stephanie **Mack**, BS ^2^, Nicole **Luisi**, PhD, MPH ^1^, Yunshu **Li**, MS ^2^, Yali **Meng**, MS ^2^, Gang **Liu**, PhD ^2^, Jiaji **Zhang**, MS ^2^, Ashly E **Jordan**, PhD, MPH ^5^, Linh **Le**, MD, MPH ^2^, Peter L **Brodie**, AEMT, BS ^6^, Svetlana **Jensen**, BS ^7^, Allan **Clear**, BS ^8^, Tomoko **Udo**, PhD ^3,9^, Vanda **Inacio**, PhD ^4^, Eli S **Rosenberg**, PhD ^2,3^

^1^ Emory University Rollins School of Public Health, Department of Epidemiology, 1518 Clifton Rd. NE, Atlanta, GA 30322

^2^ New York State Department of Health, Office of Science, Empire State Plaza, Albany, NY 12237

^3^ University at Albany, College of Integrated Health Sciences, Department of Epidemiology and Biostatistics, 1400 Washington Avenue, Albany NY 12222

^4^ School of Mathematics and Maxwell Institute for Mathematical Sciences, University of Edinburgh, James Clerk Maxwell Building, The King’s Buildings campus, Peter Guthrie Tait Road, Edinburgh, EH9 3FD, UK

^5^ New York State Office of Addiction Services and Supports, Research and Program Evaluation, New York, NY

^6^ New York State Department of Health, Division of State Emergency Medical Services, 875 Central Avenue, Albany, NY 12206

^7^ New York State Department of Health, Bureau of Narcotic Enforcement, 150 Broadway, Menands, NY 12204

^8^ New York State Department of Health, Office of Drug User Health, 90 Church Street, New York, NY 10007

^9^ University at Albany, College of Integrated Health Sciences, Department of Health Policy, Management, and Behavior, 1400 Washington Avenue, Albany NY 12222

**Table of contents**

| **Table S1**. Contingency Table: Overall Distribution of Adults, Aged 18+ with Opioid Use Disorder (OUD) across Data Sources, Including Mortality Data, New York State, Adults Aged 18+, 2020 | 1 |
| --- | --- |
| **Table S2.** Contingency Table: Overall Distribution of Adults, Aged 18+ with Opioid Use Disorder (OUD) across Data Sources, Excluding Mortality Data, New York State, Adults Aged 18+, 2020 | 1 |
| **Appendix S1.** Model Description | 2 |
| **Table S3.** Interaction Terms in Candidate Models with Posterior Inclusion Probabilities (PIPs), New York State, adults aged 18 – 65, 2020 | 5 |
| **Figure S1.** Posterior Density Estimates, Prevalence Estimates of OUD by Case Definition, New York State, Adults Aged 18 – 65, 2020 | 7 |
| **Table S4**. Capture-Recapture Estimates of Opioid Use Disorder (OUD) Prevalence by Region, Age, and Sex, New York State, Adults Aged 18+, 2020 | 8 |

**Table S1. Contingency Table: Overall Distribution of Adults, Aged 18+ with Opioid Use Disorder (OUD) across Data Sources, Including Mortality Data, New York State, Adults Aged 18+, 2020**

| SPARCS | PMP | EMS | VS | Number of OUD |
| --- | --- | --- | --- | --- |
| 1 | 0 | 0 | 0 | 44,483 |
| 0 | 1 | 0 | 0 | 58,513 |
| 1 | 1 | 0 | 0 | 17,212 |
| 0 | 0 | 1 | 0 | 5,998 |
| 1 | 0 | 1 | 0 | 4,345 |
| 0 | 1 | 1 | 0 | 1,079 |
| 1 | 1 | 1 | 0 | 2,364 |
| 0 | 0 | 0 | 1 | 3,148 |
| 1 | 0 | 0 | 1 | 323 |
| 0 | 1 | 0 | 1 | 241 |
| 1 | 1 | 0 | 1 | 138 |
| 0 | 0 | 1 | 1 | 28 |
| 1 | 0 | 1 | 1 | 41 |
| 0 | 1 | 1 | 1 | 9 |
| 1 | 1 | 1 | 1 | 20 |
|  |  |  |  | 137,942 |

**Table S2. Contingency Table: Overall Distribution of Adults, Aged 18+ with Opioid Use Disorder (OUD) across Data Sources, Excluding Mortality Data, New York State, Adults Aged 18+, 2020**

| SPARCS | PMP | EMS | Number of OUD |
| --- | --- | --- | --- |
| 1 | 0 | 0 | 44,806 |
| 0 | 1 | 0 | 58,754 |
| 1 | 1 | 0 | 17,350 |
| 0 | 0 | 1 | 6,026 |
| 1 | 0 | 1 | 4,386 |
| 0 | 1 | 1 | 1,088 |
| 1 | 1 | 1 | 2,384 |
|  |  |  | 134,794 |

**Appendix S1. Model Description**

Log-linear model

The contingency tables used for our Bayesian capture-recapture analyses consist of ${n=2}^{k}\times c_{r}\times c_{a}\times c_{g}$ cell entries, where $k$ is 3 or 4, depending on whether the death data is included, $c_{r}$, $c_{a}$, and $c_{g}$ denote the total number of groups used for the covariates region, age, and gender, respectively. For each of the contingency tables, there is a total of $c_{r}\times c_{a}\times c_{g}$ unobserved cell counts, corresponding to the number of individuals not observed by any data sources for each possible combination of covariates. Each cell count ($y_{j}$) of a contingency table with $n$ entries is assumed to be of the form:

$$y_{j}|\mu_{j}\sim\mathrm{Poisson}\left( \mu_{j} \right), j=1,\cdots,n,$$

(S1)

where $\text{log(}\mu_{j}\text{) = }{(1,\boldsymbol{x}_{j})}^{T}\boldsymbol{\beta},$ $\boldsymbol{\beta}=(\beta_{0},\beta_{1},\cdots,\beta_{q})$ is the coefficient vector of length $(q+1)$, and $\boldsymbol{x}_{j}$ represents the binary design vector of length $q$ that identifies which elements of $\beta_{1},\cdots,\beta_{q}$ are applicable to the $j$-th cell. In the coefficient vector, $\beta_{0}$ denotes the intercept, and $\beta_{1},\cdots,\beta_{q}$ corresponds to the main-effect and log-linear interaction terms. The main effect term for each data source or covariate corresponds to the influence that the source or covariate has on the underlying capture rate; the interaction terms describe the interaction between the data sources and/or covariates in terms of changing this capture rate for the given combination of sources and/or covariates. For example, a positive two-way interaction between two data sources indicates that being observed by one source increases the likelihood of being observed by the other, and vice versa.

Given (S1), the expected count corresponding to each of the unobservable cell entries $j^{*}$ is

$$\text{E}\left( y_{j^{*}} \right)= \mu_{j^{*}},$$

where $\text{log(}\mu_{j^{*}}\text{) =} {(1,\boldsymbol{x}_{j^{*}})}^{T}\boldsymbol{\beta},$ and $\boldsymbol{x}_{j^{*}}$ is the vector that takes the value 1 or 0 in the positions corresponding to the main effects of the covariates, and 0s elsewhere. The population size for each of the stratums (the combinations of the three covariates) in the table is then predicted as the sum of an unobserved cell count $\text{E}\left( y_{j^{*}} \right)$ and the observed cell counts corresponding to the stratum. The total population size is predicted as $\hat{N}=\sum_{j^{*}} \text{E}\left( y_{j^{*}} \right)+\sum_{j\neq j^{*}} y_{j}$. The population size for other specific individual covariate values, e.g., OUD population size by region, or combinations, e.g., OUD population size by region and age, can be similarly calculated.

The prediction of the unobserved cell counts relies on the parameters $\boldsymbol{\beta}$, which are estimated by fitting the observed cell counts $y_{j}$ ($j\neq j^{*})$ to the log-linear model. The inclusion of different interaction terms leads to many possible log-linear models. Model selection is hence typically required. The estimate of the population size, or the estimate of the coefficients ($\boldsymbol{\beta}$), can be sensitive to the choice of model. Instead of using a single model for population size estimation, a Bayesian model-averaging approach is considered, where both the model selection uncertainty (in terms of which interactions to be selected) and the parameter uncertainty (in terms of the coefficient estimation under a certain model) can be taken into account. We next present our Bayesian model-averaging approach.

Bayesian model-averaging

We apply a Bayes model-averaging approach^3,4^ in our analysis. In particular, we consider the set of all hierarchical log-linear models^9^, denoted by $\mathcal{M}$. Let $\boldsymbol{\beta=}\left( \beta_{0}\boldsymbol{,}\beta_{1}\boldsymbol{,\cdots,}\beta_{q} \right)$ denote the coefficient vector of length $\left( q+1 \right)$ corresponding to the maximal model (the saturated model here) in the set $\mathcal{M}$, and $\boldsymbol{\beta}_{m}=\left( \beta_{0}\boldsymbol{,}\beta_{1}\boldsymbol{,\cdots,}\beta_{q_{m}} \right)\subseteq\boldsymbol{\beta}$ denote the coefficients corresponding to the model $m\mathcal{\in M}$, and $q_{m}\leq q$. For each model, $m\mathcal{\in M}$, the posterior distribution of the coefficients $\boldsymbol{\beta}_{m}$ is given by

$$\pi\left( \boldsymbol{\beta}_{m},m | ,\boldsymbol{y} \right)\propto\pi\left( \boldsymbol{y} | \boldsymbol{\beta}_{m},m \right)\pi\left( \boldsymbol{\beta}_{m}|m \right)\pi\left( m \right),$$

where $\pi\left( \boldsymbol{y} | \boldsymbol{\beta}_{m} \right)=\prod_{j} \pi(y_{j}|\mu_{j})$ is the likelihood of (S1), and $\pi\left( \boldsymbol{\beta}_{m}|m \right)$ refers to the priors of the coefficients on the model $m$. We consider $\pi\left( \beta_{0} \right)\propto1$ and

$$\beta_{1}\boldsymbol{,\cdots,}\beta_{q}|\sigma^{2},m \sim\text{N}_{q}\left( 0,\sigma^{2}n\left( {\boldsymbol{X}_{m}}^{T}\boldsymbol{X}_{m} \right)^{-1} \right),$$

$$\sigma^{2}\sim\text{Inverse-Gamma}\left( a/2,b/2 \right),$$

(S2)

where $\boldsymbol{X}_{m}$ is the $n\times q$ matrix with rows given by $\boldsymbol{x}_{j}$, $a$ and $b$ are pre-specified hyper-parameters for the newly introduced variable $\sigma^{2}$. For our application, we follow Overstall and King^S1^ and specify a non-informative prior such that $a=1$ and $b=0$^S2^. Further, we specify a uniform prior for each model, $m$, such that $\pi\left( m \right)=1/{|\mathcal{M}|},$ where $|\mathcal{M}|$ denotes the number of models in $\mathcal{M.}$

With the prior setting (S2), the model-averaged estimate for the parameters is then given by

$$\pi\left( \boldsymbol{\beta,}\sigma^{2} | \boldsymbol{y} \right)=\sum_{m\in\mathcal{M}} \pi\left( \boldsymbol{\beta}_{m},\sigma^{2},m | \boldsymbol{y} \right),$$

where $\pi\left( \boldsymbol{\beta}_{m},\sigma^{2},m | \boldsymbol{y} \right)\propto\pi\left( \boldsymbol{y} | \boldsymbol{\beta}_{m},m \right)\pi\left( \boldsymbol{\beta}_{m}|\sigma^{2},m \right)\pi(\sigma^{2})\pi\left( m \right)$, and $\pi\left( \boldsymbol{\beta}_{m},\sigma^{2},m | \boldsymbol{y} \right)$ denotes the joint posterior probability of the model $m$ and its corresponding regression parameters, given the data, and quantitatively provides a relative comparison of competing models.

Due to the complexity of the model settings, there is unfortunately no analytical form of the posterior $\pi\left( \boldsymbol{\beta,}\sigma^{2} | \boldsymbol{y} \right)$. To explore the posterior distribution, a reversible jump Markov chain Monte Carlo (RJMCMC) algorithm^6^ was applied. The main idea of the RJMCMC is to construct a Markov chain whose invariant distribution is the desired posterior distribution, i.e., $\pi\left( \boldsymbol{\beta,}\sigma^{2} | \boldsymbol{y} \right)$, which is called the target distribution, so that draws generated by this Markov chain after equilibrium has been reached can be assumed to be from the target distribution. In summary, the RJMCMC works as follows^6^. At each iteration, given the current model $m$, with its corresponding parameters $\boldsymbol{\beta}_{m}$, a move to the model $k$ with $\boldsymbol{\beta}_{k}$ is proposed, which differs from $m$ in terms of adding one more parameter (i.e. log-linear term) that is not in $m$ or deleting one parameter from $m$. This proposal is then accepted with a probability. The posterior model probability is then estimated as the proportion of time a given model is retained in the chain. Similarly, the posterior inclusion probability (PIP) of a log-linear term can be estimated as the proportion of time a given term is retained in the models in the chain. The model-averaged estimate of the total population size, $\hat{N}$, is then computed as a weighted average of the corresponding estimated population size in each given model, where the weights are the posterior model probability of the given model.

The implementation of the Bayesian model-averaging approach was conducted via the R package *mse*^5^. For each analysis, the RJMCMC sampling algorithm was run twice, using different initialisations. A total number of 100000 iterations with a burn-in of 10000 was set for each run. A total of 180000 posterior samples from the two chains that passed the Gelman-Rubin convergence check^S3^, which checks RJMCMC convergence by assessing the similarity between multiple chains, have then been used for estimation.

In this analysis, a non-informative prior is used due to the lack of any a priori knowledge. An informative prior setting can be incorporated when such information is available. For example, informative priors on the total number of the target population^S4-S6^, as well as the a priori information on the direction and presence of the interaction terms between different data sources and/or covariates^S4^.

*********

References

S1. Overstall AM, King R. conting: An R package for Bayesian analysis of complete and incomplete contingency tables. Journal of Statistical Software. 2014;58:1-27.

S2. Gelman A. Prior distributions for variance parameters in hierarchical models (comment on article by Browne and Draper). Bayesian Analysis. 2006;1(3):515 - 534.

S3. Gelman A, Rubin DB. Inference from iterative simulation using multiple sequences. Statistical Science. 1992;7(4):457-72.

S4. King R, Bird SM, Hay G, Hutchinson SJ. Estimating current injectors in Scotland and their drug-related death rate by sex, region and age-group via Bayesian capture—recapture methods. Statistical Methods in Medical Research. 2009;18(4):341-59.

S5. King R, Bird SM, Overstall A, Hay G, Hutchinson SJ. Injecting drug users in Scotland, 2006: Listing, number, demography, and opiate-related death-rates. Addiction Research & Theory. 2013;21(3):235-46.

S6. King R, Bird SM, Overstall AM, Hay G, Hutchinson SJ. Estimating prevalence of injecting drug users and associated heroin-related death rates in England by using regional data and incorporating prior information. Journal of the Royal Statistical Society Series A: Statistics in Society. 2014;177(1):209-36.

**Table S3. Interaction Terms in Candidate Models with Posterior Inclusion Probabilities (PIPs), New York State, adults aged 18 – 65, 2020**

Posterior inclusion probability (PIP) of each unique interaction term that was included in models for each Bayesian analysis presented in Table 2, and corresponding posterior mean and standard deviation (sd), conditional on the term being present in the model. We consider a term to be included if its PIP is greater than 0.05. We consider a term to be “unique” if it is included in different analyses with different PIPs. In these analyses, to avoid the model fitting issue caused by the sparseness of some of the tables, we set the maximal model as the one that includes up to three-way interactions, encompassing interactions among lists (restricted to A1, A2, C1, and C2), among covariates, and between lists and covariates.

|  |  | **Case Definition 1 ^a^** | | |  | **Case Definition 2 ^a^** | | |
| --- | --- | --- | --- | --- | --- | --- | --- | --- |
| **Methodological Component** |  | **Interaction term** | **Posterior inclusion probability (PIP)** | **Mean (sd)** |  | **Interaction term** | **Posterior inclusion probability (PIP)** | **Mean (sd)** |
| Data Deduplication Not Applied  Overdose Mortality Data Inclusion | A1 | DTH:Age | 0.07 | 0.01(0.04) | A2 | DTH:Age | 0.95 | -0.3(0.11) |
|  |  | DTH:Region | 0.99 | 0.2(0.08) |  | DTH:Region | 1 | 0.26(0.06) |
|  |  | PMP:DTH | 0.87 | 0.56(0.37) |  | PMP:DTH | 1 | 0.15(0.12) |
|  |  | EMS:DTH | 1 | 2.61(0.35) |  | EMS:DTH | 1 | 1.2(0.27) |
|  |  | PMP:DTH:Region | 0.72 | 0.29(0.21) |  | PMP:DTH:Region | 1 | 0.54(0.12) |
|  |  | EMS:DTH:Region | 0.41 | 0.12(0.16) |  | EMS:DTH:Region | 0.64 | 0.28(0.23) |
|  |  | EMS:DTH:Sex | 0.79 | 0.28(0.16) |  | EMS:DTH:Sex | 0.25 | 0.09(0.17) |
|  |  | SPARCS:EMS:Region | 0.54 | 0.07(0.07) |  | SPARCS:EMS:Region | 1 | 0.28(0.06) |
|  |  | SPARCS:EMS:DTH | 1 | -1.09(0.3) |  | SPARCS:EMS:DTH | 0.89 | 0.43(0.21) |
|  |  | SPARCS:PMP:Region | 0.25 | 0.03(0.06) |  |  |  |  |
|  |  | PMP:EMS:Region | 1 | 0.49(0.05) |  |  |  |  |
|  |  | SPARCS:PMP:Age | 1 | 0.28(0.08) |  |  |  |  |
|  |  | SPARCS:PMP:EMS | 0.57 | 0.33(0.32) |  |  |  |  |
|  |  | SPARCS:PMP:DTH | 0.82 | 0.58(0.37) |  |  |  |  |
| Data Deduplication Not Applied  Overdose Mortality Data Exclusion | B1 | SPARCS:PMP:Region | 0.75 | 0.11(0.07) | B2 | SPARCS:PMP:Region | 0.74 | 0.25(0.2) |
|  |  | SPARCS:EMS:Region | 0.18 | 0.02(0.05) |  | SPARCS:EMS:Region | 0.93 | 0.39(0.18) |
|  |  | PMP:EMS:Region | 1 | 0.49(0.05) |  | PMP:EMS:Region | 0.19 | 0.03(0.07) |
| Data Deduplication Applied ^b^ Overdose Mortality Data Inclusion | C1 | DTH:Age | 0.14 | 0.03(0.09) | C2 | DTH:Age | 0.98 | 0.33(0.08) |
|  |  | DTH:Region | 0.12 | 0.01(0.05) |  | DTH:Region | 0.08 | 0.01(0.04) |
|  |  | PMP:DTH | 0.1 | 0(0.06) |  | PMP:DTH | 0.22 | 0.03(0.13) |
|  |  | EMS:DTH | 1 | 1.47(0.17) |  | EMS:DTH | 0.3 | 0.19(0.31) |
|  |  | PMP:DTH:Region | 0.06 | 0.01(0.08) |  | PMP:DTH:Region | 0.06 | 0.02(0.09) |
|  |  | EMS:DTH:Sex | 0.79 | 0.36(0.21) |  | EMS:DTH:Sex | 0.21 | -0.19(0.34) |
|  |  | SPARCS:EMS:Region | 0.37 | 0.06(0.09) |  | SPARCS:EMS:Region | 1 | 0.62(0.05) |
|  |  | SPARCS:PMP:Region | 0.93 | 0.24(0.07) |  | SPARCS:PMP:Region | 0.95 | 0.26(0.06) |
|  |  | SPARCS:PMP:Age | 1 | -0.2(0.09) |  | SPARCS:PMP:Age | 0.07 | 0.01(0.06) |
|  |  | PMP:EMS:Region | 1 | 0.33(0.05) |  |  |  |  |
|  |  |  |  |  |  | SPARCS:PMP:DTH | 0.13 | 0.03(0.11) |
|  |  |  |  |  |  | SPARCS:PMP:EMS | 0.83 | 0.36(0.14) |
| Data Deduplication Applied ^b^ Overdose Mortality Data Exclusion | D1 | SPARCS:PMP:Region | 0.98 | 0.33(0.07) | D2 | SPARCS:PMP:Region | 1 | 0.53(0.09) |
|  |  | SPARCS:EMS:Region | 0.06 | 0.01(0.05) |  | SPARCS:EMS:Region | 0.12 | 0.02(0.07) |
|  |  | PMP:EMS:Region | 1 | 0.29(0.05) |  | PMP:EMS:Region | 0.74 | 0.13(0.09) |
|  |  |  |  |  |  |  |  |  |
| a See Table 1 for Data sources and Definitions | | | | | | | | |
| b Deduplication: Between SPARCS/EMS for the same encounter: removed cases where individual only captured as OUD in SPARCS if transported to hospital/ED within 2 days after OUD EMS encounter; Persons with OUD indicated as dead in EMS or SPARCS, and also found in Vital Records OUD data were removed from Vital Records and only counted once in EMS or SPARCS wherever they were first observed. | | | | | | | | |

**Figure S1. Posterior Density Estimates, Prevalence Estimates of OUD by Case Definition, New York State, Adults Aged 18 – 65, 2020**

Posterior density estimates of the prevalence estimate of OUD for case definition 1 (blue) and case definition 2 (green) for methodological practices (a [top left]) all source lists; (b [top right]) SPARCS, PMP, EMS; (c [bottom left]) SPARCS, EMS, and VS de-duplicated for referrals, PMP; and (d [bottom right]) SPARCS and EMS de-duplicated for referrals, PMP. A bimodality is observed for A1 in Figure (a), due to different models that include or exclude the term SPARCS:PMP:EMS being identified in the analysis, and hence resulted in a larger 95% CI.

**Table S4. Capture-Recapture Estimates of Opioid Use Disorder (OUD) Prevalence by Region, Age, and Sex, New York State, Adults Aged 18+, 2020**

|  | | | **Observed OUD** | **Unobserved OUD** | **Total Estimate OUD** | | | **Prevalence Estimate OUD** | | | **Population Size** |
| --- | --- | --- | --- | --- | --- | --- | --- | --- | --- | --- | --- |
| **Region** | **Age** | **Sex** | **n** | **n** | **n** | **95% CI** | | **%** | **95% CI** | |  |
|  |  |  |  |  |  | **lower** | **upper** |  | **lower** | **upper** |  |
| Total | 18-29 | Male | 13,023 | 35,111 | 48,134 | 44,540 | 52,024 | 2.93% | 2.71% | 3.16% | 1,644,958 |
|  |  | Female | 8,106 | 21,297 | 29,404 | 26,860 | 32,208 | 1.80% | 1.64% | 1.97% | 1,634,611 |
|  |  | Total | 21,129 | 56,408 | 77,538 | 72,255 | 83,241 | 2.36% | 2.20% | 2.54% | 3,279,569 |
|  | 30-64 | Male | 66,629 | 243,325 | 309,954 | 289,161 | 332,257 | 7.09% | 6.61% | 7.59% | 4,374,772 |
|  |  | Female | 36,795 | 145,370 | 182,165 | 169,132 | 196,209 | 3.97% | 3.68% | 4.27% | 4,590,616 |
|  |  | Total | 103,424 | 388,695 | 492,119 | 459,099 | 527,511 | 5.49% | 5.12% | 5.88% | 8,965,388 |
|  | 65+ | Male | 6,473 | 37,863 | 44,335 | 38,882 | 50,595 | 3.19% | 2.80% | 3.65% | 1,387,793 |
|  |  | Female | 3,768 | 30,265 | 34,033 | 28,312 | 40,553 | 1.85% | 1.54% | 2.21% | 1,836,934 |
|  |  | Total | 10,241 | 68,128 | 78,369 | 70,316 | 87,360 | 2.43% | 2.18% | 2.71% | 3,224,727 |
|  | Total | Male | 86,125 | 316,299 | 402,424 | 375,204 | 431,548 | 5.43% | 5.07% | 5.83% | 7,407,523 |
|  |  | Female | 48,669 | 196,932 | 245,602 | 227,816 | 265,076 | 3.05% | 2.83% | 3.29% | 8,062,161 |
| NYC | 18-29 | Male | 3,087 | 9,343 | 12,430 | 11,214 | 13,812 | 1.73% | 1.56% | 1.92% | 720,514 |
|  |  | Female | 1,206 | 4,411 | 5,617 | 4,900 | 6,450 | 0.74% | 0.64% | 0.85% | 761,532 |
|  |  | Total | 4,293 | 13,754 | 18,047 | 16,342 | 19,981 | 1.22% | 1.10% | 1.35% | 1,482,046 |
|  | 30-64 | Male | 24,637 | 87,099 | 111,736 | 103,390 | 121,173 | 5.94% | 5.50% | 6.44% | 1,880,987 |
|  |  | Female | 9,648 | 41,635 | 51,283 | 46,811 | 56,280 | 2.51% | 2.30% | 2.76% | 2,039,103 |
|  |  | Total | 34,285 | 128,734 | 163,019 | 150,821 | 176,755 | 4.16% | 3.85% | 4.51% | 3,920,090 |
|  | 65+ | Male | 3,632 | 12,594 | 16,226 | 14,296 | 18,471 | 3.14% | 2.76% | 3.57% | 517,118 |
|  |  | Female | 1,614 | 8,002 | 9,616 | 7,903 | 11,642 | 1.30% | 1.07% | 1.58% | 736,868 |
|  |  | Total | 5,246 | 20,596 | 25,842 | 22,931 | 29,206 | 2.06% | 1.83% | 2.33% | 1,253,986 |
|  | Total | Male | 31,356 | 109,036 | 140,392 | 130,076 | 152,017 | 4.50% | 4.17% | 4.87% | 3,118,619 |
|  |  | Female | 12,468 | 54,048 | 66,517 | 60,832 | 72,908 | 1.88% | 1.72% | 2.06% | 3,537,503 |
|  |  | Total | 43,824 | 163,084 | 206,908 | 191,669 | 223,976 | 3.11% | 2.88% | 3.36% | 6,656,122 |
| ROS | 18-29 | Male | 9,936 | 25,768 | 35,704 | 32,819 | 38,836 | 3.86% | 3.55% | 4.20% | 924,444 |
|  |  | Female | 6,900 | 16,886 | 23,786 | 21,712 | 26,076 | 2.72% | 2.49% | 2.99% | 873,079 |
|  |  | Total | 16,836 | 42,654 | 59,490 | 55,128 | 64,196 | 3.31% | 3.07% | 3.57% | 1,797,523 |
|  | 30-64 | Male | 41,992 | 156,226 | 198,218 | 183,046 | 214,343 | 7.95% | 7.34% | 8.60% | 2,493,785 |
|  |  | Female | 27,147 | 103,735 | 130,882 | 120,627 | 142,032 | 5.13% | 4.73% | 5.57% | 2,551,513 |
|  |  | Total | 69,139 | 259,961 | 329,100 | 304,397 | 355,406 | 6.52% | 6.03% | 7.04% | 5,045,298 |
|  | 65+ | Male | 2,841 | 25,269 | 28,110 | 23,941 | 32,957 | 3.23% | 2.75% | 3.79% | 870,675 |
|  |  | Female | 2,154 | 22,263 | 24,417 | 20,006 | 29,487 | 2.22% | 1.82% | 2.68% | 1,100,066 |
|  |  | Total | 4,995 | 47,532 | 52,527 | 46,183 | 59,678 | 2.67% | 2.34% | 3.03% | 1,970,741 |
|  | Total | Male | 54,769 | 207,263 | 262,032 | 241,915 | 283,532 | 6.11% | 5.64% | 6.61% | 4,288,904 |
|  |  | Female | 36,201 | 142,884 | 179,085 | 164,792 | 194,606 | 3.96% | 3.64% | 4.30% | 4,524,658 |
|  |  | Total | 90,970 | 350,147 | 441,117 | 408,102 | 476,354 | 5.00% | 4.63% | 5.40% | 8,813,562 |
| Overall | - | - | 134,794 | 513,232 | 648,026 | 604,744 | 694,752 | 4.19% | 3.91% | 4.49% | 15,469,684 |

Abbreviations: NYC, New York City; NYS, New York State; ROS, rest of state.
